# The Lentiviral Integrase Binding Protein LEDGF/p75 and HIV-1 Replication

**DOI:** 10.1371/journal.ppat.1000046

**Published:** 2008-03-28

**Authors:** Alan Engelman, Peter Cherepanov

**Affiliations:** 1 Department of Cancer Immunology and AIDS, Dana-Farber Cancer Institute, Division of AIDS, Harvard Medical School, Boston, Massachusetts, United States of America; 2 Division of Medicine, Imperial College London, St. Mary's Campus, London, United Kingdom; University of British Columbia, Canada

## Abstract

Retroviral replication proceeds through a stable proviral DNA intermediate, and numerous host cell factors have been implicated in its formation. In particular, recent results have highlighted an important role for the integrase-interactor lens epithelium-derived growth factor (LEDGF)/p75 in lentiviral integration. Cells engineered to over-express fragments of LEDGF/p75 containing its integrase-binding domain but lacking determinants essential for chromatin association are refractory to HIV-1 infection. Furthermore, both the levels of HIV-1 integration and the genomic distribution of the resultant proviruses are significantly perturbed in cells devoid of endogenous LEDGF/p75 protein. A strong bias towards integration along transcription units is a characteristic feature of lentiviruses. In the absence of LEDGF/p75, HIV-1 in large part loses that preference, displaying concomitant integration surges in the vicinities of CpG islands and gene promoter regions, elements naturally targeted by other types of retroviruses. Together, these findings highlight that LEDGF/p75 is an important albeit not strictly essential cofactor of lentiviral DNA integration, and solidify a role for chromatin-associated LEDGF/p75 as a receptor for lentiviral preintegration complexes. By now one of the best characterized virus–host interactions, the integrase-LEDGF/p75 interface opens a range of opportunities for lentiviral vector targeting for gene therapy applications as well as for the development of novel classes of antiretroviral drugs.

## Introduction

**Figure 1:**
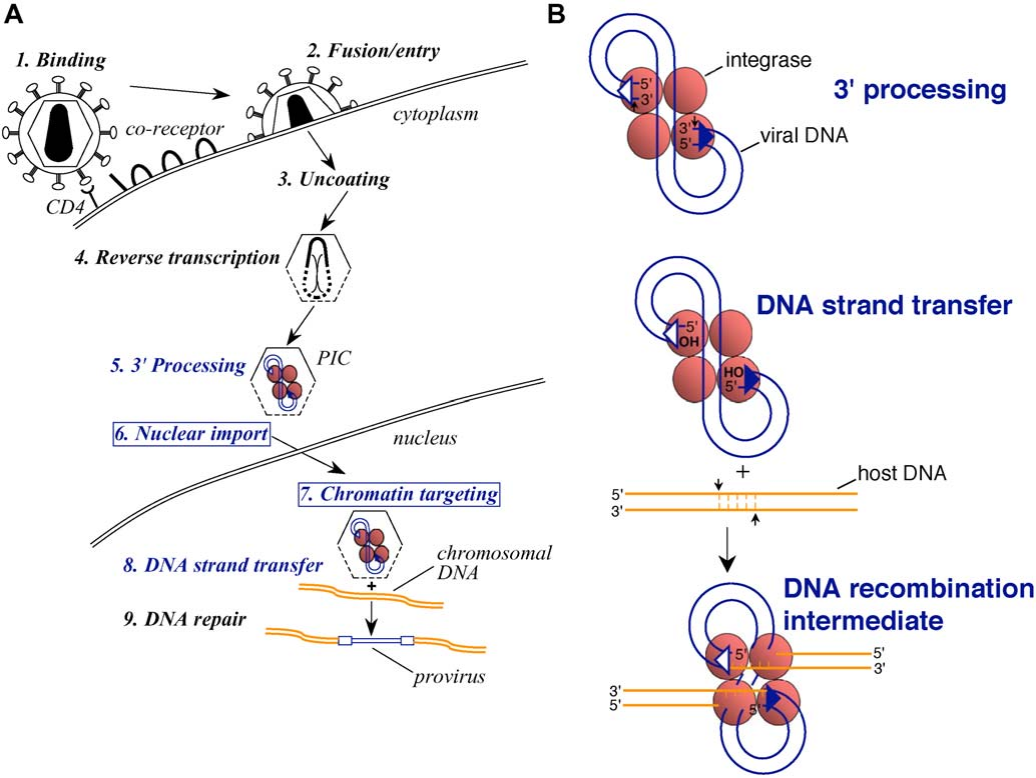
Retroviral Early Events and the Mechanism of HIV-1 DNA Integration. (A) The early phase of the retroviral lifecycle is divided into nine steps, spanning from step 1, when the extracellular virus initially engages its cellular receptor, to step 9, with the completion of provirus formation. Steps 5–8, which encompass IN 3′ processing of the nascent reverse transcript in the cytoplasm to the formation of the DNA strand transfer reaction product in the nucleus (highlighted in blue font), could potentially be affected by the IN–LEDGF/p75 interaction. Among these steps, lentiviruses display unique nuclear import and chromatin targeting properties (blue boxes). (B) Details of phosphodiester bond breakage and joining during HIV-1 integration. Small vertical arrows denote the bonds cleaved by IN, using water as the chemical nucleophile for 3′ processing, and the OH groups at the 3′ ends of the processed viral DNA for DNA strand transfer [4]. Results of in vitro experiments indicate that a dimer of HIV-1 IN suffices to process each viral DNA end, whereas a tetramer is required for DNA strand transfer activity [92]–[94]. IN is known to function as a multimer during infection [95],[96]; notably, though, its functional PIC-associated multimeric form has not been determined. A tetramer is represented at each step here for simplicity. The open and filled triangles at the ends of the viral DNA represent U3 and U5 sequences, respectively, important for IN function (reviewed in [97]). Host cell factors are likely to repair the single strand gaps present within the DNA recombination intermediate (reviewed in [5]).

A key step in the retroviral lifecycle is the formation of the provirus, the integrated form of the viral cDNA that is produced during reverse transcription. Retroviral integration is promoted by the viral integrase (IN) enzyme, which enters the cell as a component of the virion particle. IN catalyzes two spatially and temporally distinct reactions within the context of the preintegration complex (PIC), a large structure derived from the virus core [1],[2]. During the initial reaction, which is called 3′ processing and happens soon after the cDNA is made, IN hydrolyzes a dinucleotide from each end of HIV-1 DNA [2],[3] (Figure 1). The second reaction, DNA strand transfer, takes place at the site of integration in the cell nucleus. IN uses the recessed 3′-OH groups created during 3′ processing to cut opposing strands of chromosomal DNA in a staggered fashion, concomitantly connecting the viral DNA 3′ ends to the generated 5′ overhangs [4]. The resultant DNA recombination intermediate harbors single-strand discontinuities that must be repaired to complete provirus formation (Figure 1). See [5] for a thorough overview of the mechanism of HIV-1 integration as well as the host cell factors that are implicated in the final DNA repair step.

Retroviral IN enzymes purified from a variety of sources display 3′ processing and DNA strand transfer activities in vitro [6]–[10]. These seminal results highlighted that IN alone sufficed to form DNA strand transfer reaction products; however, numerous subsequent studies indicated that other proteins play important auxiliary roles in the context of virus infection (see [5], [11]–[13] for recent reviews). This review focuses on the IN interactor lens epithelium-derived growth factor (LEDGF)/transcriptional co-activator p75, whose critical role in lentiviral DNA integration has been highlighted by a number of recent studies [14]–[18].

## LEDGF/p75: A Cellular Interactor of Lentiviral INs

LEDGF/p75, a member of the hepatoma-derived growth factor (HDGF) related protein (HRP) family, was initially implicated in lentiviral biology through its association with ectopically expressed HIV-1 IN in 293T cells [19]. Significantly, purified recombinant LEDGF/p75 protein stimulated HIV-1 IN catalytic function in vitro [19]. The interaction was independently discovered by analyzing proteins associated with HIV-1 IN in HeLa cells [5] and in a yeast two-hybrid screen for HIV-1 IN interactors [20].

**Figure 2:**
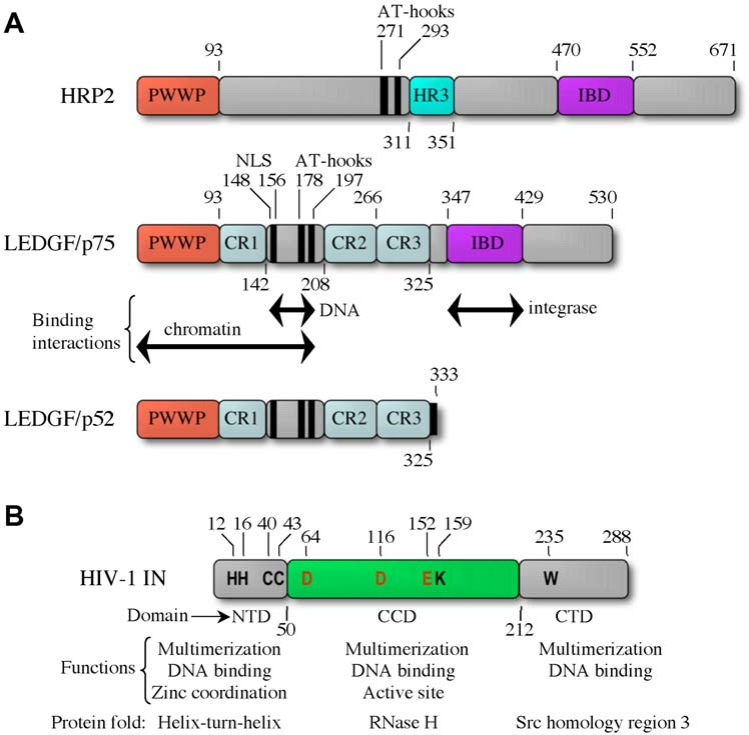
Domain Organization of LEDGF/p75 and HIV-1 IN Proteins. (A) LEDGF/p75 and related proteins. The binding of LEDGF/p75 to DNA in vitro is mediated by the NLS and a nearby dual copy of the AT-hook DNA binding motif [31], whereas the N-terminal PWWP domain supplies a critical chromatin recognition function [31],[32]. Charged regions (CRs) 1–3 work in concert with the PWWP domain and AT-hooks to affect the wild-type chromatin binding phenotype as determined by biochemical fractionation [32]. The function of the HRP2 AT-hooks, identifiable though sequence gazing [25], has not been established experimentally. A patch of conserved amino acids known as homology region III (HR3) was also identified via aligning multiple HRP2 orthologs [25]. The LEDGF/p75 IBD is critical for stimulation of HIV-1 IN function in vitro [25],[31],[51],[77],[78],[98] and for HIV-1 infection [16],[18]. The N-terminal 325 residues within LEDGF/p75 and LEDGF/p52 are identical, whereas the p52 isoform harbors a unique 8–amino acid residue tail [27]. (B) The three IN domains as defined by a number of structural and functional studies are shown (refer to [5] and [13] for details). Amino acid residues within each domain that are conserved across *Retroviridae* are indicated. The Asp and Glu residues highlighted in red within the CCD coordinate Mg ions for catalysis during 3′ processing and DNA strand transfer.

HRPs are characterized by a conserved N-terminal PWWP domain, an ∼90– to 135–amino acid module found in a variety of nuclear proteins [21],[22]. Six human HRP family members have been described: HDGF, HRP1, HRP2, HRP3, LEDGF/p75, and LEDGF/p52 [23]–[25], of which two, LEDGF/p75 and HRP2, possess affinity for HIV-1 IN [25]. Significantly larger than the rest of the HRPs, LEDGF/p75 and HRP2 contain a second evolutionarily conserved domain within their extended C-termini. It is this domain that mediates the interaction with HIV-1 IN, hence the term “IN-binding domain (IBD)” [25],[26] (Figure 2A). LEDGF/p75 and LEDGF/p52 are expressed from the same gene (human *PSIP1*) [27]. The smaller p52 isoform, produced by alternative RNA splicing [27], lacks the IBD and fails to engage HIV-1 IN in vitro or in live cells [28] (Figure 2A).

LEDGF/p75 is a ubiquitous nuclear protein, tightly associated with chromatin throughout the cell cycle [19], [26], [28]–[31]. Chromatin association is primarily mediated by three conserved sequence elements within the N-terminal half of the protein: the PWWP domain, nuclear localization signal (NLS), and a dual copy of the AT-hook DNA binding motif [31],[32] (Figure 2A). LEDGF/p75 binds a variety of DNA substrates in vitro, an activity that appears relevant to its association with chromatin [31]. We note that the sequence-specific DNA binding of LEDGF/p75 to stress response and heat shock elements [33] could not be independently verified [31]. Recent results have revealed that the association with chromatin is essential for LEDGF/p75 function during HIV-1 infection ([16],[18]; see below), highlighting the importance of clarifying the mechanism of LEDGF/p75 chromatin binding.

The cellular functions of LEDGF/p75 and closely related HRP2 remain largely uncharacterized, although initial reports have indicated a role for LEDGF/p75 in transcriptional regulation [27],[33],[34]. The protein is not essential for cell survival [18], though the majority of LEDGF-null mice died soon after birth or showed a range of developmental abnormalities in adulthood [35]. Of note, the eye lens developed normally in LEDGF knockout mice [35], highlighting that its most often used name, which was coined during the second isolation of the gene [36], reflects the use of lens epithelial cells for cDNA isolation more so than biological function. The finding that LEDGF/p75 associates with JPO2, a known interactor of c-MYC, will hopefully help efforts to link its function to an established cellular mechanism [37],[38].

HIV-1 and feline immunodeficiency virus (FIV) INs predominantly localize to nuclei upon ectopic expression in a variety of cell types [30], [39]–[42]. In mitotic cells, and likely throughout the cell cycle, the lentiviral INs stably associate with chromatin [30],[40],[41]. Three important consequences on IN expression occurred when levels of endogenous LEDGF/p75 protein were reduced using RNA interference (RNAi). First, in interphase cells, the vast majority of HIV-1 and FIV IN re-localized to the cell cytoplasm [28],[30]. Second, and perhaps more significant, IN proteins lost their chromosomal association, as was most clearly observed in mitotic cells [28],[30]. Finally, at least in case of HIV-1 IN, the redistribution was accompanied by significant reductions in steady-state levels of the viral protein [43]. These observations implicated LEDGF/p75 as the dominant cellular interactor of lentiviral INs and indicated that the cellular protein was essential for IN-chromatin association, likely acting as a receptor or molecular tether. LEDGF/p75 thus contributes to the karyophilic properties of lentiviral INs. Indeed, the cell factor contains a classical NLS (residues 148–156; Figure 2A) [26],[44],[45], and over-expression of a NLS defective form of LEDGF/p75 trapped HIV-1 IN in the cytoplasm [26],[44]. However, the viral protein has not been formally proven to piggyback into the nucleus through its interaction with LEDGF/p75. In one report, a primarily nuclear IN population was observed when knockdown cells were treated with proteasome inhibitors [20]. Although the association of ectopically expressed HIV-1 IN with chromatin is attributable to the LEDGF/p75 interaction, these data suggest the viral protein might access nuclei in a LEDGF/p75-independent manner. The mechanisms of lentiviral IN/PIC nuclear import remain ongoing areas of investigation (see [46],[47] for recent reviews).

Additional studies revealed that LEDGF/p75 binds to a variety of lentiviral IN proteins, but, significantly, fails to interact with IN proteins derived from five (α, β, δ, γ, and Spuma) of the six other tested retroviral genera [30],[48],[49]. These observations implied that the IN-LEDGF/p75 interaction underlies a unique aspect of lentiviral biology. Of note, lentiviral PICs access cell nuclei and target chromatin (Figure 1A, steps 6 and 7) using mechanisms that distinguish them from other retroviruses [46],[47],[50].

## Structural Aspects of the IN–LEDGF/p75 Interaction

HIV-1 IN is composed of three functional domains: the N-terminal domain (NTD), the catalytic core domain (CCD), and the C-terminal domain (CTD) (Figure 2B). Initial mapping experiments using fluorescent fusions expressed in live cells revealed that the CCD is minimally required for the interaction with LEDGF/p75, and highlighted a role for the NTD as an affinity enhancer [28]. A number of single amino acid substitutions within the CCD, including V165A [5], R166A [51], and Q168A [20], were soon thereafter shown to impair the IN–LEDGF/p75 interaction. Each of these changes rendered HIV-1 replication defective [20], [52]–[54], suggesting that the IN–LEDGF/p75 interaction might be essential for HIV-1 replication [20],[51],[55]. However, many mutations in IN exert so-called “class II” pleiotropic effects, whereby poorly understood aspects of IN biology extending beyond its innate catalytic function contribute to the overall replication defect [55]–[57]. Recent results indicate that PICs formed in the absence of LEDGF/p75 protein in vivo are fully competent to integrate the endogenous cDNA made during reverse transcription into exogenous target DNA in vitro [18]. Based on this, one would predict that IN mutant viruses defective for growth solely due to the inability to interact with LEDGF/p75 would yield PICs fully competent for integration in vitro. The replication defect caused by the Q168A mutation was suggested to result from the lack of cofactor binding [20], though a follow-up study indicated HIV-1_Q168A_ behaved as a class II IN mutant virus [55]. PICs derived from class II mutants fail to support in vitro integration activity [58], indicating that PIC analyses would help to shed light on the specificity of the HIV-1_Q168A_ replication defect.

**Figure 3:**
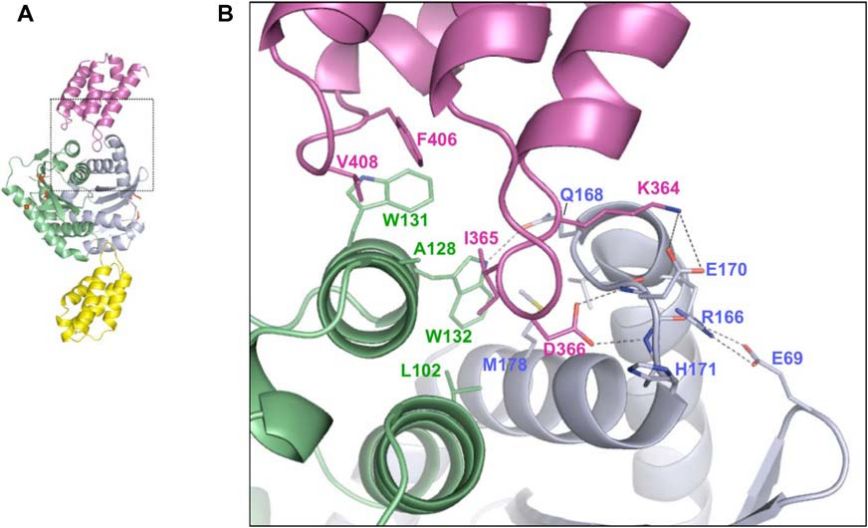
Crystal Structure of the LEDGF/p75-IN Interaction. (A) Cartoon representation of the CCD-IBD complex (the complete asymmetric unit) [60]. IN CCD molecules are colored green and blue, whereas the LEDGF/p75 IBDs are magenta and yellow. The side chains of IN active site residues Asp-64, Asp-116, and Glu-152 (Figure 2B) are shown as red sticks. The region within the dashed box is expanded in (B). (B) Details of the CCD-IBD interface. LEDGF/p75 hotspot residues Ile-365 and Asp-366, situated at the base of the loop between IBD helices 1 and 2, project into a pocket at the CCD dimer interface. The bidentate hydrogen bond contact between Asp-366 and the backbone amides of IN residues Glu-170 and His-171 is critical for the protein–protein interaction in vitro [51], in yeast cells [55], and during HIV-1 infection [18]. Ile-365 is buried into a hydrophobic pocket predominantly formed by IN residues Ala-128, Trp-132, Leu-102, and Met-178. Hydrogen bonds and salt bridges are shown as dotted lines. The figure was drawn using PyMOL [99].

The 3-D structure of the LEDGF/p75 IBD solved by nuclear magnetic resonance spectroscopy revealed a compact α-helical domain possessing topological and structural similarities to HEAT repeat domains [51]. The HEAT repeat, an α-helical hairpin module containing 37–47 amino acid residues, is a versatile building block found among diverse protein families, and derives its name from Huntingtin, elongation factor 3, the regulatory subunit of protein phosphatase 2A, and PI3-kinase TOR [59]. Whereas HEAT repeat proteins typically contain numerous individual hairpins, the LEDGF/p75 IBD is comprised of only two repeats and was therefore classified as a pseudo-HEAT repeat analogous topology (PHAT) domain [51]. Substituting Ala for either Ile-365, Asp-366, or Phe-406 ablated the IN–LEDGF/p75 interaction in vitro, defining these amino acids as hotspot contact residues [51]. Their principal involvement in the protein–protein interaction was confirmed through determination of the crystal structure of the IBD in complex with the IN CCD [60] (Figure 3). The LEDGF/p75 binding site on IN notably forms via tertiary structural interactions, as the IBD burrows into a cleft created by the IN dimer interface [60] (Figure 3A). The side chain carbonyl of LEDGF/p75 Asp-366 forms a bidentate hydrogen bond with the backbone amides of residues Glu-170 and His-171 from one IN monomer, while Ile-365 and Phe-406 participate in multiple hydrophobic interactions with residues primarily donated from the other monomer. In particular, the side chain of Ile-365 becomes buried within a hydrophobic pocket (Figure 3B). Substituting Asn for Asp-366 ablated the IN–LEDGF/p75 interaction in vitro [51] and in yeast cells [55], highlighting the central role of the bidentate hydrogen bond in the interaction. Several critical contacts with the IN polypeptide backbone explain the mechanism whereby LEDGF/p75 binds to a variety of lentiviral proteins, although the INs fail to reveal recognizable sequence conservation at these amino acid positions [49],[60]. The main chain traces of other retroviral INs would appear to differ significantly at key points of IBD–CCD contact, accounting for the lentiviral specificity of the LEDGF/p75–IN interaction [60].

## LEDGF/p75 and HIV-1 Replication

Initial RNAi-based studies were central to establish important links between endogenous LEDGF/p75 protein and lentiviral IN expression levels and subcellular localization, yet they failed to reveal an important role for the cell factor in HIV-1 replication. In some RNAi-based studies, despite achieving what appeared to be very efficient reductions in cellular protein [30],[61], specific HIV-1 replication defects were not observed despite rigorous effort to identify them. At the time it was suggested that intracellular LEDGF/p75 levels might significantly exceed those required to effect normal lentiviral DNA integration [61], a hypothesis supported by subsequent RNAi-based work [16],[62],[63] and a gene knockout study [18]. Llano and colleagues [16] performed an elegant study whereby the expression of short-hairpin RNA (shRNA) was linked to that of green fluorescence protein (GFP) within the same lentiviral-based vector. Sorting the brightest green cells therefore ensured for selection of potent LEDGF/p75 knockdowns. Selected cells were moreover fractionated to analyze levels of chromatin-bound protein. In this way, HIV-1 infectivity levels were correlated to residual levels of chromatin-associated LEDGF/p75. In the absence of detectable protein, infection was reduced to 3.5% of that observed in the presence of normal LEDGF/p75 levels. Similarly significantly reduced levels of HIV-1 infection were observed in mouse embryo fibroblasts (MEFs) derived from LEDGF knockout as compared to littermate control animals [18]. The block in both cases was at integration: reverse transcription and the formation of two long terminal repeat (LTR)–containing DNA circles, a surrogate marker for PIC nuclear import, were normal, whereas integration was severely reduced [16],[18]. Although these results would seem to exclude a role for LEDGF/p75 in the nuclear import of the PIC, the baffling ability of lentiviruses to infect non-dividing cell types with high efficiency calls for further scrutiny of lentiviral PIC nuclear import in LEDGF-depleted cells under conditions of growth arrest. It is important to stress that even in a genetic knockout model completely devoid of LEDGF/p75 protein, HIV-1 integration was not ablated: LEDGF-null MEFs supported ∼10% of the level of HIV-1 integration achieved in control cells [18]. Hence, although important for lentiviral integration, LEDGF/p75 is clearly not strictly essential. The infectivity of Moloney murine leukemia virus (Mo-MLV), a γ-retrovirus, importantly did not depend on the presence of LEDGF/p75 in target cells [16],[18], providing biological relevance to the observations of lentiviral specificity in the IN–LEDGF/p75 interaction.

HIV-1 infection was fully restored to LEDGF/p75-depleted cells by ectopic expression of the cell factor [16],[18],[62], allowing detailed mutational analyses of cofactor function. Mutagenesis studies highlighted two regions within LEDGF/p75, the IBD and N-terminal PWWP domain/AT-hook DNA binding motifs that mediate chromatin binding (Figure 2A), as crucial for HIV-1 infection [16],[18]. The requirement for the N-terminal sequence elements lent credence to early conjectures that LEDGF/p75 might primarily function to tether HIV-1 PICs to chromatin for integration [19],[28],[30].

The isolated IBD competitively inhibited LEDGF/p75-dependent stimulation of IN activity in vitro [25], and GFP-IBD fusion proteins over-expressed in target cells rather potently restricted HIV-1 infection [15],[16]. The inhibitory effect was specific, since Mo-MLV was not affected, and altering IBD hotspot residue Asp-366 negated the block to HIV-1 infection. Importantly, reverse transcription and nuclear localization proceeded normally, with a significant reduction in total HIV-1 DNA levels observed after approximately six cell divisions. These data were fully consistent with an integration block, though the lentiviral vectors used to create cell lines precluded direct measurements of HIV-1 integration [15]. These observations indicate that IBD binding in large part precluded IN from engaging chromatin-associated LEDGF/p75. It is noteworthy that IBD over-expression, when combined with shRNA-mediated depletion, yielded a multiplicative antiviral effect [16] that would appear to exceed that observed in cells completely devoid of LEDGF/p75 protein [18]. It seems plausible that IBD-bound IN is crippled in its capacity to effectively engage target DNA in the absence of effective levels of competing chromosomal LEDGF/p75.

An HIV-1 mutant selected for its ability to replicate in MT-4 T cells engineered to express the C-terminal portion of the p75 isoform (residues 326–530; Figure 2A) acquired two mutations in IN, A128T and E170G [17]. Predictably, the mutations affected residues whose side chains directly participate in the LEDGF/p75-binding interface. As illustrated by the IBD-CCD crystal structure, Glu-170 is involved in a salt bridge with Lys-364 of LEDGF/p75, whereas Ala-128 contributes to the hydrophobic pocket that buries LEDGF/p75 hotspot residue Ile-365 [60] (Figure 3B). As expected, these mutations reduced the apparent affinity of the interaction with LEDGF/p75 [17]. Altering IN residue Ala-128 to the bulkier Gln had been previously shown to reduce the affinity of the protein–protein interaction [55]. Intriguingly, HIV-1_A128T/E170G_ was partially defective,and its replication capacity was reduced further upon LEDGF/p75 knockdown, suggesting that the mutant IN still depended on LEDGF/p75 for integration [17]. The estimated K_d_ of the interaction between wild type HIV-1 IN and LEDGF/p75 is notably in the low nM range (PC and AE, unpublished data). Conceivably, by slightly detuning the LEDGF/p75-binding interface, the escape mutations afforded the dissociation of PIC-born IN from non-productive complex formation with the LEDGF/p75 fragment. Following multiple cycles of association/dissociation, the PIC would eventually engage a functional cofactor molecule.

These results provided physiological evidence for previous contentions that LEDGF/p75 is the dominant cellular binding partner of HIV-1 IN. Indeed, cell factors that potentially bind to other regions of IN were clearly unable to compensate for the loss in LEDGF/p75 binding caused by the A128T/E170G mutations. One interpretation is that LEDGF/p75 is the only cell protein involved in tethering HIV-1 to chromosomes for integration [17], though it remains plausible that other factors that bind IN with lower affinity could play roles in this process. As the ability of HRP2 to stimulate HIV-1 IN activity in vitro was inhibited by an excess of the LEDGF/p75 IBD fragment, both HRP protein family members are predicted to bind IN in similar manners [25]. Over-expression of human HRP2 in LEDGF-null cells rescued HIV-1 infection [18], although the analogous experiment failed to reveal a significant infectivity boost in severely knocked-down human SupT1 cells [16]. The generation of mouse HRP2 knockout cells, currently under way in the Engelman laboratory, should help to clarify whether this IBD-containing protein plays a significant role in HIV-1 integration.

## LEDGF/p75 and PIC Targeting during Lentiviral Integration

Establishment of the stably integrated provirus is a hallmark of retroviral replication, fundamental to the persistence of infection. However, mammalian genomic DNA is a vast target, a significant proportion of which is not transcriptionally active. What's more, when integrated, the transcriptional activity of viral cDNA becomes sensitive to the local chromosomal environment [64]. Hence, it is not surprising that retroviruses do not leave integration entirely to chance, having evolved mechanisms for selecting suitable target loci. Indeed, the observed distributions of integrated proviruses along host chromosomal DNA are not random, and biases at the level of local DNA sequences as well as on the genomic scale have been described (reviewed in [13] and [50]). These biases appear to be genus-specific, and although the differences are sometimes subtle, three retroviral genera appear to stand out most distinctly. Lentiviruses, including HIV (both type 1 and type 2) [65],[66], simian immunodeficiency virus (SIV) [67],[68], FIV [69], and equine infectious anemia virus (EIAV) [70], are strongly biased towards integration into transcription units (TUs), with a preference for highly expressed genes. The γ-retrovirus Mo-MLV, in contrast to lentiviruses, prefers to integrate in the vicinity of transcription start sites and CpG islands [67],[71], while a spumaretrovirus, simian foamy virus (SFV), is biased *against* integrating into genes, yet nonetheless displays significant preferences for gene start sites and CpG islands [72],[73].

Disengagement of HIV-1 IN from chromatin upon knockdown of endogenous cellular LEDGF/p75 strongly suggested that the cofactor acts as a chromosomal receptor or molecular tether for IN [28],[30]. In addition, the interaction with LEDGF/p75 is conserved among and unique to *Lentivirus* [30],[48],[49], essentially paralleling the genus' notable bias towards integration into TUs. The anticipation that LEDGF/p75 is the lentiviral targeting factor was initially confirmed by Ciuffi et al., who reported statistically significant albeit modest reductions of HIV-1 integration into TUs in two of three human cell lines following LEDGF/p75 knockdown [14]. More recently, a novel genetic knockout model afforded the study of HIV-1 integration under LEDGF-null conditions [18]. The observed frequency of HIV-1 integration into TUs in LEDGF knockout cells was significantly lower than in the littermate control condition. Importantly, the selectivity of HIV-1 for TUs in LEDGF-null cells was marginally less than the levels observed for γ- and α-retroviruses, as well as the adeno-associated parvovirus, in normal human cells [18]. Integration distribution and frequency positively correlated with local transcription activity in the absence of LEDGF/p75, though this correlation was significantly reduced compared with cells expressing the host factor [18]. The frequency of HIV-1 integration in the vicinity of CpG islands and transcription start sites was augmented in the absence of LEDGF/p75 [18], yielding profiles more similar to α-, γ-, δ-, and spuma-retroviruses, which naturally display affinity for these genomic features [71]–[76]. These data suggest that *Retroviridae* might exploit a common mechanism for intranuclear trafficking and integration site selection, which lentiviruses have evolved to override by the virtue of their interaction with LEDGF/p75. These findings solidified that LEDGF/p75 is the principal *Lentivirus*-specific integration targeting factor [18]. Nevertheless, in our minds, it is impossible to rule out that other cellular factors would contribute to the observed integration site distribution.

## The Model for LEDGF/p75 Function in HIV-1 Integration

**Figure 4:**
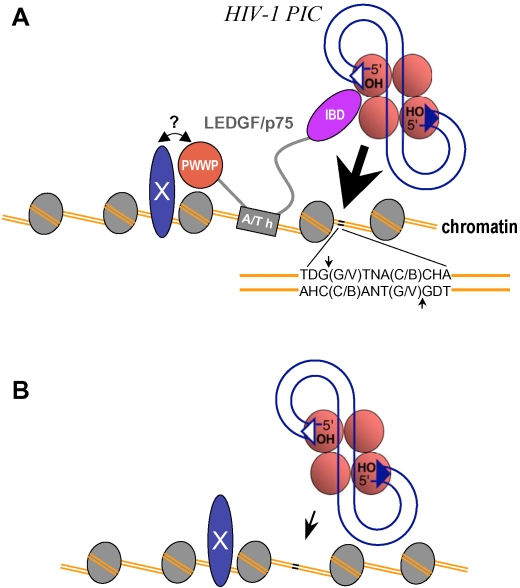
Model of LEDGF/p75 Function during HIV-1 Integration. (A) The basic unit of chromatin, the nucleosome, is depicted as a gray oval of histone proteins in association with chromosomal DNA (orange lines). LEDGF/p75 might engage chromatin via its NLS and AT-hook motifs (A/T h) binding directly to DNA [31] and/or by the PWWP domain and AT-hooks mediating protein interactions with histone proteins or other currently unknown chromatin factors (labeled X) [18],[31],[32]. Upon engaging the IBD, PIC-bound IN is encouraged to integrate the viral cDNA at a nearby position, statistically favoring the indicated palindromic target DNA sequence (indicated by short black lines, and expanded to denote the sequence) [18], [82]–[85]. The short vertical lines indicate the sites of integration on the two chromosomal DNA strands. (B) The PIC can still engage the consensus target DNA sequence in the absence of LEDGF/p75, though overall levels of HIV-1 integration are reduced ∼10-fold under this condition (represented by the relative size of the arrow in [A] and [B]) [18].

The question of how important LEDGF/p75 is to HIV-1 replication has caused fierce debates and remained controversial until recently. On one hand, viral replication defects caused by mutations in and around the LEDGF/p75-binding interface of HIV-1 IN indicated that the cofactor might play an essential role [20],[51],[55]. On the other hand, LEDGF/p75 depletion via RNAi significantly reduced but fell short of abrogating HIV-1 integration [16],[62],[63]. Since LEDGF knockout cells supported residual levels of HIV-1 integration, we must conclude that the cofactor is not essential for integration [18]. We concede that it is possible that some additional mechanisms rescue HIV-1 integration in the mouse knockout system, or that murine HRP2 takes over the role of LEDGF/p75. Nevertheless, these results are in agreement with a large body of experimental evidence demonstrating that the isolated HIV-1 IN protein can perform its catalytic functions [7],[10] (reviewed in [5] and [13]). Furthermore, PICs assembled in LEDGF-null cells were fully functional in vitro, indicating that the cofactor is not essential for the assembly or intrinsic activity of the HIV-1 complex. Though the endogenous cellular protein readily co-immunoprecipitated lentiviral PICs [30], the equivalent activities of PICs isolated from normal and LEDGF-null cells strongly suggest that it is the chromatin-bound pool of the protein that is functionally relevant (Figure 4).

In our model, chromatin-associated LEDGF/p75 acts as a receptor for incoming PIC particles, and although the PICs possess the capacity to engage target DNA, the interaction with LEDGF/p75 encourages IN's DNA strand transfer activity, thereby directing integration to a nearby genomic locus (Figure 4A). Concordantly, the cofactor robustly stimulates the enzymatic activities of lentiviral INs in vitro [19],[25],[31],[49],[77],[78]. The model suggests that when the pool of chromatin-associated LEDGF/p75 is reduced or ablated, a larger fraction of PICs will rely on a slower, cofactor-independent pathway (Figure 4B). Conceivably, this will extend the time a PIC lingers in the non-integrated state, widening the window of opportunity for the cell to destroy it, and hence affecting overall integration levels. Indeed, the nucleoprotein complexes generated during reverse transcription [79],[80] as well as IN itself [41],[43],[81] are subject to degradation by the cellular ubiquitin-proteasome system. The model accounts for the retention of the weakly favored target DNA consensus sequence at the sites of HIV-1 integration [82]–[85] in the absence of LEDGF/p75 [18], and also explains why a detectable shift in the distribution of HIV-1 integration sites occurred under partial LEDGF/p75 knockdown conditions that nonetheless were insufficiently weak to affect the overall level of virus infection [14].

## Conclusions and Perspectives

LEDGF/p75 is an important host factor commandeering the integration of HIV-1 and likely other lentiviruses to active TUs [14],[18]. Although LEDGF/p75 was required for efficient HIV-1 integration and replication [62],[63], it is not essential, since stringently knocked-down human SupT cells and knockout MEFs supported residual provirus formation (approximately 10% of that seen in the presence of endogenous LEDGF/p75 levels) [16],[18]. Therefore, it appears that HIV-1 does not entirely rely on the host factor, and furthermore, it can be assumed that there is a background of LEDGF/p75-independent integration under normal infection conditions. Conceivably, integration into transcriptionally repressed or gene-poor regions contributes to the establishment of latent viral reservoirs and hence to the persistence of clinical infection [86]. It remains to be determined if the residual levels of integration in LEDGF-null cells depend upon HRP2, a close kin of LEDGF/p75.

The potential for directed integration could alleviate concerns of insertional mutagenesis and in theory greatly improve the safety of retroviral vectors in gene therapy applications. Given the central role it plays in directing lentiviral integration [14],[18] and its modular structural organization [25], LEDGF/p75 seems to be an ideal candidate for creating a designer targeting factor for lentiviral vectors. Indeed, a protein chimera containing the LEDGF/p75 IBD and the DNA-binding domain of bacteriophage λ repressor stimulated HIV-1 integration in the vicinity of λ operator sequences in vitro [87]. Undoubtedly, the future will see more work in this important direction. Also impending is focus on clarifying the cellular functions of LEDGF/p75 and the related HRPs. Broad changes (both increases and decreases) in the transcriptional activity of ∼2,000 genes were observed in silenced human 293T cells [14], with significantly fewer (<200) genes affected in LEDGF-null MEFs [18]. Although the chromosomal distribution of LEDGF/p75 has not been addressed directly, based on well-established HIV-1 integration site preferences [85], it is expected to be associated with a large body of active genes, distributed along the length of TUs [18],[74]. A function in RNA polymerase II transcription elongation or splicing would agree with this distribution pattern.

The significant reductions in HIV-1 infectivity observed in cells extensively depleted for LEDGF/p75 protein [16],[18] and in those engineered to over-express IBD-containing fragments [15]–[17], as well as the remarkable conservation of the interaction among divergent lentiviruses [49], highlight the protein–protein interaction as a novel target for the development of antiviral drugs. Though protein–protein interactions are traditionally more challenging than enzyme active sites for targeted drug development [88], LEDGF/p75 hotspot residues Ile-356 and Asp-366 notably extend down into a cleft formed at the IN dimer interface (Figure 3B). It is not difficult to envision a small molecule with sufficient binding affinity occupying the pocket and precluding LEDGF/p75 from binding. A major hurdle in identifying such a compound will be the strength of LEDGF/p75–IN interaction, although recent results seem to suggest this may not be insurmountable. A compound that ablated HIV-1 IN 3′ processing and DNA strand transfer activities in vitro at the relatively modest median inhibition concentration (IC_50_) of 150 µM bound the CCD at the IBD interaction site [60],[89]. This region of IN is therefore amenable to small molecule binding. Peptides derived from the face of the IBD that interacts with the CCD were moreover reported to inhibit IN activity in a noncompetitive manner [90]. At present, development of clinically useful small molecules possessing sufficient affinity to preclude LEDGF/p75 binding and inhibit HIV-1 replication remains an inspiring challenge.

After the acceptance of this Review, Marshall et al. [91] reported that the frequency and distribution of HIV-1 as well as equine infectious anemia virus integration were significantly altered in human cells knocked down for LEDGF/p75 expression and in mouse cells carrying disrupted *Psip1* sequences.

## Supporting Information

### 

Accession Numbers

#### 

Detailed information on the following genes and proteins can be accessed at the National Center for Biotechnology Information (http//:www.ncbi.nlm.nih.gov/) using the following accession numbers: HIV‐1 IN (NP_705928), LEDGF/p75 (NP_150091), HDGF (NP_004485), HRP1 (NP_612641), HRP2 (NP_001001520), HRP3 (NP_057157), LEDGF/p52 (NP_066967), *PSIP1* (GeneID 11168); JPO2 (Q96GN5), c‐MYC (NP_002458), and FIV IN (NP_040973). The Protein Data Bank (http://www.rcsb.org/pdb) accession number for the CCD‐IBD complex is 2B4J.

## References

[1] Bowerman B, Brown PO, Bishop JM, Varmus HE (1989). A nucleoprotein complex mediates the integration of retroviral DNA.. Genes Dev.

[2] Miller M, Farnet C, Bushman F (1997). Human immunodeficiency virus type 1 preintegration complexes: Studies of organization and composition.. J Virol.

[3] Pauza C (1990). Two bases are deleted from the termini of HIV-1 linear DNA during integrative recombination.. Virology.

[4] Engelman A, Mizuuchi K, Craigie R (1991). HIV-1 DNA integration: Mechanism of viral DNA cleavage and DNA strand transfer.. Cell.

[5] Turlure F, Devroe E, Silver PA, Engelman A (2004). Human cell proteins and human immunodeficiency virus DNA integration.. Front Biosci.

[6] Katzman M, Katz RA, Skalka AM, Leis J (1989). The avian retroviral integration protein cleaves the terminal sequences of linear viral DNA at the in vivo sites of integration.. J Virol.

[7] Bushman FD, Fujiwara T, Craigie R (1990). Retroviral DNA integration directed by HIV integration protein in vitro.. Science.

[8] Craigie R, Fujiwara T, Bushman F (1990). The IN protein of Moloney murine leukemia virus processes the viral DNA ends and accomplishes their integration in vitro.. Cell.

[9] Katz RA, Merkel G, Kulkosky J, Leis J, Skalka AM (1990). The avian retroviral IN protein is both necessary and sufficient for integrative recombination in vitro.. Cell.

[10] Sherman PA, Fyfe JA (1990). Human immunodeficiency virus integration protein expressed in Escherichia coli possesses selective DNA cleaving activity.. Proc Natl Acad Sci U S A.

[11] Ciuffi A, Bushman FD (2006). Retroviral DNA integration: HIV and the role of LEDGF/p75.. Trends Genet.

[12] Van Maele B, Busschots K, Vandekerckhove L, Christ F, Debyser Z (2006). Cellular co-factors of HIV-1 integration.. Trends Biochem Sci.

[13] Vandegraaff N, Engelman A (2007). Molecular mechanism of HIV integration and therapeutic intervention.. Expert Rev Mol Med.

[14] Ciuffi A, Llano M, Poeschla E, Hoffmann C, Leipzig J (2005). A role for LEDGF/p75 in targeting HIV DNA integration.. Nat Med.

[15] De Rijck J, Vandekerckhove L, Gijsbers R, Hombrouck A, Hendrix J (2006). Overexpression of the lens epithelium-derived growth factor/p75 integrase binding domain inhibits human immunodeficiency virus replication.. J Virol.

[16] Llano M, Saenz DT, Meehan A, Wongthida P, Peretz M (2006). An essential role for LEDGF/p75 in HIV integration.. Science.

[17] Hombrouck A, De Rijck J, Hendrix J, Vandekerckhove L, Voet A (2007). Virus evolution reveals an exclusive role for LEDGF/p75 in chromosomal tethering of HIV.. PLoS Pathog.

[18] Shun M-C, Raghavendra NK, Vandegraaff N, Daigle JE, Hughes S (2007). LEDGF/p75 functions downstream from preintegration complex formation to effect gene-specific HIV-1 integration.. Genes Dev.

[19] Cherepanov P, Maertens G, Proost P, Devreese B, Van Beeumen J (2003). HIV-1 integrase forms stable tetramers and associates with LEDGF/p75 protein in human cells.. J Biol Chem.

[20] Emiliani S, Mousnier A, Busschots K, Maroun M, Van Maele B (2005). Integrase mutants defective for interaction with LEDGF/p75 are impaired in chromosome tethering and HIV-1 replication.. J Biol Chem.

[21] Stec I, Nagl SB, van Ommen G-JB, den Dunnen JT (2000). The PWWP domain: A potential protein-protein interaction domain in nuclear proteins influencing differentiation?. FEBS Letters.

[22] Qiu C, Sawada K, Zhang X, Cheng X (2002). The PWWP domain of mammalian DNA methyltransferase Dnmt3b defines a new family of DNA-binding folds.. Nat Struct Biol.

[23] Izumoto Y, Kuroda T, Harada H, Kishimoto T, Nakamura H (1997). Hepatoma-derived growth factor belongs to a gene family in mice showing significant homology in the amino terminus.. Biochem Biophys Res Commun.

[24] Ikegame K, Yamamoto M, Kishima Y, Enomoto H, Yoshida K (1999). A new member of a hepatoma-derived growth factor gene family can translocate to the nucleus.. Biochem Biophys Res Commun.

[25] Cherepanov P, Devroe E, Silver PA, Engelman A (2004). Identification of an evolutionarily conserved domain in LEDGF/p75 that binds HIV-1 integrase.. J Biol Chem.

[26] Vanegas M, Llano M, Delgado S, Thompson D, Peretz M (2005). Identification of the LEDGF/p75 HIV-1 integrase-interaction domain and NLS reveals NLS-independent chromatin tethering.. J Cell Sci.

[27] Ge H, Si Y, Roeder RG (1998). Isolation of cDNAs encoding novel transcription coactivators p52 and p75 reveals an alternate regulatory mechanism of transcriptional activation.. EMBO J.

[28] Maertens G, Cherepanov P, Pluymers W, Busschots K, De Clercq E (2003). LEDGF/p75 is essential for nuclear and chromosomal targeting of HIV-1 integrase in human cells.. J Biol Chem.

[29] Nishizawa Y, Usukura J, Singh DP, Chylack LTJ, Shinohara T (2001). Spatial and temporal dynamics of two alternatively spliced regulatory factors, lens epithelium-derived growth factor (ledgf/p75) and p52, in the nucleus.. Cell Tissue Res.

[30] Llano M, Vanegas M, Fregoso O, Saenz D, Chung S (2004). LEDGF/p75 determines cellular trafficking of diverse lentiviral but not murine oncoretroviral integrase proteins and is a component of functional lentiviral preintegration complexes.. J Virol.

[31] Turlure F, Maertens G, Rahman S, Cherepanov P, Engelman A (2006). A tripartite DNA-binding element, comprised of the nuclear localization signal and two AT-hook motifs, mediates the association of LEDGF/p75 with chromatin *in vivo*.. Nucleic Acids Res.

[32] Llano M, Vanegas M, Hutchins N, Thompson D, Delgado S (2006). Identification and characterization of the chromatin-binding domains of the HIV-1 integrase interactor LEDGF/p75.. J Mol Biol.

[33] Singh DP, Fatma N, Kimura A, Chylack J, Leo T, Shinohara T (2001). LEDGF binds to heat shock and stress-related element to activate the expression of stress-related genes.. Biochem Biophys Res Commun.

[34] Fatma N, Singh DP, Shinohara T, Chylack LT (2001). Transcriptional regulation of the antioxidant protein 2 gene, a thiol-specific antioxidant, by lens epithelium-derived growth factor to protect cells from oxidative stress.. J Biol Chem.

[35] Sutherland HG, Newton K, Brownstein DG, Holmes MC, Kress C (2006). Disruption of Ledgf/Psip1 results in perinatal mortality and homeotic skeletal transformations.. Mol Cell Biol.

[36] Singh DP, Ohguro N, Chylack LT, Shinohara T (1999). Lens epithelium-derived growth factor: increased resistance to thermal and oxidative stresses.. Invest Ophthalmol Vis Sci.

[37] Maertens GN, Cherepanov P, Engelman A (2006). Transcriptional co-activator p75 binds and tethers the c-Myc-interacting protein JPO2 to chromatin.. J Cell Sci.

[38] Bartholomeeusen K, De Rijck J, Busschots K, Desender L, Gijsbers R (2007). Differential interaction of HIV-1 integrase and JPO2 with the C terminus of LEDGF/p75.. J Mol Biol.

[39] Pluymers W, Cherepanov P, Schols D, De Clercq E, Debyser Z (1999). Nuclear localization of human immunodeficiency virus type 1 integrase expressed as a fusion protein with green fluorescent protein.. Virology.

[40] Cherepanov P, Pluymers W, Claeys A, Proost P, De Clercq E (2000). High-level expression of active HIV-1 integrase from a synthetic gene in human cells.. FASEB J.

[41] Devroe E, Engelman A, Silver PA (2003). Intracellular transport of human immunodeficiency virus type 1 integrase.. J Cell Sci.

[42] Woodward CL, Wang Y, Dixon WJ, Htun H, Chow SA (2003). Subcellular localization of feline immunodeficiency virus integrase and mapping of its karyophilic determinant.. J Virol.

[43] Llano M, Delgado S, Vanegas M, Poeschla EM (2004). Lens epithelium-derived growth factor/p75 prevents proteasomal degradation of HIV-1 integrase.. J Biol Chem.

[44] Maertens G, Cherepanov P, Debyser Z, Engelborghs Y, Engelman A (2004). Identification and characterization of a functional nuclear localization signal in the HIV-1 integrase interactor LEDGF/p75.. J Biol Chem.

[45] Singh DP, Kubo E, Takamura Y, Shinohara T, Kumar A (2006). DNA binding domains and nuclear localization signal of LEDGF: Contribution of two helix-turn-helix (HTH)-like domains and a stretch of 58 amino acids of the N-terminal to the trans-activation potential of LEDGF.. J Mol Biol.

[46] Fassati A (2006). HIV infection of non-dividing cells: A divisive problem.. Retrovirology.

[47] Suzuki Y, Craigie R (2007). The road to chromatin—nuclear entry of retroviruses.. Nat Rev Microbiol.

[48] Busschots K, Vercammen J, Emiliani S, Benarous R, Engelborghs Y (2005). The interaction of LEDGF/p75 with integrase is lentivirus-specific and promotes DNA binding.. J Biol Chem.

[49] Cherepanov P (2007). LEDGF/p75 interacts with divergent lentiviral integrases and modulates their enzymatic activity in vitro.. Nucleic Acids Res.

[50] Bushman F, Lewinski M, Ciuffi A, Barr S, Leipzig J (2005). Genome-wide analysis of retroviral DNA integration.. Nat Rev Microbiol.

[51] Cherepanov P, Sun ZY, Rahman S, Maertens G, Wagner G (2005). Solution structure of the HIV-1 integrase-binding domain in LEDGF/p75.. Nat Struct Mol Biol.

[52] Wiskerchen M, Muesing MA (1995). Human immunodeficiency virus type 1 integrase: Effects of mutations on viral ability to integrate, direct viral gene expression from unintegrated viral DNA templates, and sustain viral propagation in primary cells.. J Virol.

[53] Limón A, Devroe E, Lu R, Ghory HZ, Silver PA (2002). Nuclear localization of human immunodeficiency virus type 1 preintegration complexes (PICs): V165A and R166A are pleiotropic integrase mutants primarily defective for integration, not PIC nuclear import.. J Virol.

[54] Bouyac-Bertoia M, Dvorin JD, Fouchier RAM, Jenkins Y, Meyer BE (2001). HIV-1 infection requires a functional integrase NLS.. Mol Cell.

[55] Rahman S, Lu R, Vandegraaff N, Cherepanov P, Engelman A (2007). Structure-based mutagenesis of the integrase-LEDGF/p75 interface uncouples a strict correlation between in vitro protein binding and HIV-1 fitness.. Virology.

[56] Engelman A (1999). In vivo analysis of retroviral integrase structure and function.. Adv Virus Res.

[57] Lu R, Limón A, Devroe E, Silver PA, Cherepanov P (2004). Class II integrase mutants with changes in putative nuclear localization signals are primarily blocked at a post-nuclear entry step of human immunodeficiency virus type 1 replication.. J Virol.

[58] Lu R, Vandegraaff N, Cherepanov P, Engelman A (2005). Lys-34, dispensable for integrase catalysis, is required for preintegration complex function and human immunodeficiency virus type 1 replication.. J Virol.

[59] Andrade MA, Petosa C, O'Donoghue SI, Muller CW, Bork P (2001). Comparison of ARM and HEAT protein repeats.. J Mol Biol.

[60] Cherepanov P, Ambrosio ALB, Rahman S, Ellenberger T, Engelman A (2005). From the Cover: Structural basis for the recognition between HIV-1 integrase and transcriptional coactivator p75.. Proc Natl Acad Sci U S A.

[61] Vandegraaff N, Devroe E, Turlure F, Silver PA, Engelman A (2006). Biochemical and genetic analyses of integrase-interacting proteins lens epithelium-derived growth factor (LEDGF)/p75 and hepatoma-derived growth factor related protein 2 (HRP2) in preintegration complex function and HIV-1 replication.. Virology.

[62] Vandekerckhove L, Christ F, Van Maele B, De Rijck J, Gijsbers R (2006). Transient and stable knockdown of the integrase cofactor LEDGF/p75 reveals its role in the replication cycle of human immunodeficiency virus.. J Virol.

[63] Zielske SP, Stevenson M (2006). Modest but reproducible inhibition of human immunodeficiency virus type 1 infection in macrophages following LEDGFp75 silencing.. J Virol.

[64] Lewinski MK, Bisgrove D, Shinn P, Chen H, Hoffmann C (2005). Genome-wide analysis of chromosomal features repressing human immunodeficiency virus transcription.. J Virol.

[65] Schroder ARW, Shinn P, Chen H, Berry C, Ecker JR (2002). HIV-1 integration in the human genome favors active genes and local hotspots.. Cell.

[66] MacNeil A, Sankale JL, Meloni ST, Sarr AD, Mboup S (2006). Genomic sites of human immunodeficiency virus type 2 (HIV-2) integration: Similarities to HIV-1 in vitro and possible differences in vivo.. J Virol.

[67] Hematti P, Hong BK, Ferguson C, Adler R, Hanawa H (2004). Distinct genomic integration of MLV and SIV vectors in primate hematopoietic stem and progenitor cells.. PLoS Biol.

[68] Crise B, Li Y, Yuan C, Morcock DR, Whitby D (2005). Simian immunodeficiency virus integration preference is similar to that of human immunodeficiency virus type 1.. J Virol.

[69] Kang Y, Moressi CJ, Scheetz TE, Xie L, Tran DT (2006). Integration site choice of a feline immunodeficiency virus vector.. J Virol.

[70] Hacker CV, Vink CA, Wardell TW, Lee S, Treasure P (2006). The integration profile of EIAV-based vectors.. Mol Ther.

[71] Wu X, Li Y, Crise B, Burgess SM (2003). Transcription start regions in the human genome are favored targets for MLV integration.. Science.

[72] Nowrouzi A, Dittrich M, Klanke C, Heinkelein M, Rammling M (2006). Genome-wide mapping of foamy virus vector integrations into a human cell line.. J Gen Virol.

[73] Trobridge GD, Miller DG, Jacobs MA, Allen JM, Kiem H-P (2006). Foamy virus vector integration sites in normal human cells.. Proc Natl Acad Sci U S A.

[74] Mitchell RS, Beitzel BF, Schroder ARW, Shinn P, Chen H (2004). Retroviral DNA integration: ASLV, HIV, and MLV show distinct target site preferences.. PLoS Biol.

[75] Narezkina A, Taganov KD, Litwin S, Stoyanova R, Hayashi J (2004). Genome-wide analyses of avian sarcoma virus integration sites.. J Virol.

[76] Derse D, Crise B, Li Y, Princler G, Lum N (2007). HTLV-1 integration target sites in the human genome: Comparison with other retroviruses.. J Virol.

[77] Pandey KK, Sinha S, Grandgenett DP (2007). Transcriptional coactivator LEDGF/p75 modulates human immunodeficiency virus type 1 integrase-mediated concerted integration.. J Virol.

[78] Yu F, Jones GS, Hung M, Wagner AH, MacArthur HL (2007). HIV-1 integrase preassembled on donor DNA is refractory to activity stimulation by LEDGF/p75.. Biochemistry.

[79] Schwartz O, Marechal V, Friguet B, Arenzana-Seisdedos F, Heard J-M (1998). Antiviral activity of the proteasome on incoming human immunodeficiency virus type 1.. J Virol.

[80] Butler SL, Johnson EP, Bushman FD (2002). Human immunodeficiency virus cDNA metabolism: Notable stability of two-long terminal repeat circles.. J Virol.

[81] Mulder LC, Muesing MA (2000). Degradation of HIV-1 integrase by the N-end rule pathway.. J Biol Chem.

[82] Carteau S, Hoffmann C, Bushman F (1998). Chromosome structure and human immunodeficiency virus type 1 cDNA integration: Centromeric alphoid repeats are a disfavored target.. J Virol.

[83] Holman AG, Coffin JM (2005). Symmetrical base preferences surrounding HIV-1, avian sarcoma/leukosis virus, and murine leukemia virus integration sites.. Proc Natl Acad Sci U S A.

[84] Wu X, Li Y, Crise B, Burgess SM, Munroe DJ (2005). Weak palindromic consensus sequences are a common feature found at the integration target sites of many retroviruses.. J Virol.

[85] Wang GP, Ciuffi A, Leipzig J, Berry CC, Bushman FD (2007). HIV integration site selection: Analysis by massively parallel pyrosequencing reveals association with epigenetic modifications.. Genome Res.

[86] Bisgrove D, Lewinski M, Bushman F, Verdin E (2005). Molecular mechanisms of HIV-1 proviral latency.. Expert Rev Anti Infect Ther.

[87] Ciuffi A, Diamond TL, Hwang Y, Marshall HM, Bushman FD (2006). Modulating target site selection during human immunodeficiency virus DNA integration in vitro with an engineered tethering factor.. Hum Gene Ther.

[88] Gonzalez-Ruiz D, Gohlke H (2006). Targeting protein-protein interactions with small molecules: Challenges and perspectives for computational binding epitope detection and ligand finding.. Curr Med Chem.

[89] Molteni V, Greenwald J, Rhodes D, Hwang Y, Kwiatkowski W (2001). Identification of a small-molecule binding site at the dimer interface of the HIV integrase catalytic domain.. Acta Crystallogr D.

[90] Hayouka Z, Rosenbluh J, Levin A, Loya S, Lebebdiker M (2007). Inhibiting HIV-1 integrase by shifting its oligomerization equilibrium.. Proc Natl Acad Sci U S A.

[91] Marshall HM, Ronen K, Berry C, Llano M, Sutherland H (2007). Role of PSIP1/LEDGF/p75 in lentiviral infectivity and integration targeting.. PLoS ONE.

[92] Faure A, Calmels C, Desjobert C, Castroviejo M, Caumont-Sarcos A (2005). HIV-1 integrase crosslinked oligomers are active in vitro.. Nucleic Acids Res.

[93] Guiot E, Carayon K, Delelis O, Simon F, Tauc P (2006). Relationship between the oligomeric status of HIV-1 integrase on DNA and enzymatic activity.. J Biol Chem.

[94] Li M, Mizuuchi M, Burke TRJ, Craigie R (2006). Retroviral DNA integration: reaction pathway and critical intermediates.. EMBO J.

[95] Fletcher TM, Soares MA, McPhearson S, Hui H, Wiskerchen M (1997). Complementation of integrase function in HIV-1 virions.. EMBO J.

[96] Wu X, Liu H, Xiao H, Conway JA, Hunter E (1997). Functional RT and IN incorporated into HIV-1 particles independently of the Gag/Pol precursor protein.. EMBO J.

[97] Katzman M, Katz RA (1999). Substrate recognition by retroviral integrases.. Adv Virus Res.

[98] Raghavendra NK, Engelman A (2007). LEDGF/p75 interferes with the formation of synaptic nucleoprotein complexes that catalyze full-site HIV-1 DNA integration in vitro: implications for the mechanism of viral cDNA integration.. Virology.

[99] DeLano WL (2002). The PyMOL molecular graphics system.. http://www.pymol.org/.

